# Barriers to Care in Veterinary Services: Lessons Learned From Low-Income Pet Guardians' Experiences at Private Clinics and Hospitals During COVID-19

**DOI:** 10.3389/fvets.2021.764753

**Published:** 2021-10-20

**Authors:** Amy Morris, Haorui Wu, Celeste Morales

**Affiliations:** ^1^Vancouver Humane Society, Vancouver, BC, Canada; ^2^School of Social Work, Dalhousie University, Halifax, NS, Canada

**Keywords:** low-income pet guardians, low-cost veterinary services, financial limitations, COVID-19, human-animal bond, one welfare

## Abstract

This qualitative study aimed to explore the experiences of low-income pet guardians in accessing veterinary care during COVID-19. Participants were recruited through a purposive sampling method: 12 individuals who applied to and met the low-income threshold to access support for veterinary fees from the Vancouver Humane Society (VHS) were invited for semi-structured in-depth telephone interviews. Participants indicated that they experienced pandemic-related barriers related to and compounded by their low-income status. Their experiences fit into three categories: the barriers to accessing veterinary care pre-and peri-COVID-19, the emotional impact of compounding barriers related to accessing veterinary care during COVID-19, and the human-animal bond and resilience in the context of COVID-19. Drawing on the One Health, One Welfare approach, we argue that veterinary and animal services should evaluate and improve their support services, particularly programs developed for low-income pet guardians. Based on the participants' recommendations, we propose that veterinary and animal services prepare for future disaster situations by increasing their financial capacity to support people needing assistance, undergoing training to better work with people experiencing financial and emotional stress, and providing easily accessible resources to better distribute knowledge about animal needs and available financial assistance programming. The suggestions are intended to benefit animals, their guardians, and both veterinary and animal service sector workers.

## Introduction

Pets provide significant diverse benefits to their guardians, particularly to those experiencing vulnerabilities (1–3). The COVID-19 pandemic has resulted in many people experiencing mental health challenges, including fears about economic consequences and traumatic stress (4). COVID-19 has also demonstrated the importance of pets in aiding in the resilience of their guardians (5). For example, Ikeuchi and colleagues highlight that during the COVID-19 pandemic, socially isolated older adults without dogs were more likely to report lower psychological health than their peers who have or have had a dog in their life (6). Furthermore, animals have been shown to positively impact how people react, cope, and recover from disaster situations (7–11). Consequently, current COVID-19-specific research reports increased pet guardianship (12) and confirms the various support roles of the animal within human-animal bonds (13).

One Health and One Welfare frameworks demonstrate the interconnections among human, co-inhabitants, and their environment (14). The One Health approach recognizes that human health is closely connected to animals and our shared environment (15). The One Welfare framework extends the approach of the One Health framework, promoting the links of animal welfare to human welfare and the environment (16). In practice, One Welfare aims to improve animal welfare and human well-being and vice versa (17). One Welfare highlights how relationships between companion animals and humans contribute to well-being. Research suggests that a healthy human-animal relationship can lead to positive physical, emotional, and social outcomes impacts, especially for vulnerable people experiencing mental health challenges (1, 14). Additionally, animal guardians experiencing vulnerabilities have strong bonds to their animals, who motivate positive behavior change in their guardians. The One Welfare approach can include identifying the mutual benefits of the human-animal bond and demonstrating how improving services can acknowledge and help preserve these bonds (1).

COVID-19 has impacted low-income guardians and their pets by compounding financial and emotional stress factors, specifically in accessing veterinary care (13). Indeed, pandemic-specific public health restrictions forced animal hospitals to cancel or limit appointments, prevented pet guardians from accompanying their pets in the clinics, and reduced some pet guardian's communication with veterinarians. This shift to curbside services potentially increased the guardian's emotional stress (18). Although these COVID-19 changes likely impacted many animal guardians, the impacts exacerbated the barriers to veterinary care that people experiencing low income already experience (19).

In a Canada-based study exploring the relationships between human social deprivation and animal surrender to shelters, Ly et al. (20) discuss the importance of the need for free or low-cost veterinary care and desexing services in low-socioeconomic status areas. Specifically, using quantitative data comparison methods, they formed recommendations that services be made available to guardians and the animals they care for to reduce the risk of surrender due to deprivation factors. These include ethnocultural composition, economic dependency, residential instability, and situational vulnerability. Increased access to veterinary care in underserved populations can help reduce animal overpopulation, improve animal welfare, and benefit overall community health from a One Health and One Welfare perspective (21).

Recent research highlights the importance of accessibility, communication, empathy, and cultural competence when low-income pet guardians seek veterinary services, specifically in accessing free and low-cost community veterinary services (22). Briefly, cultural competence is defined as awareness, behaviors, knowledge, attitudes, skills, and policies that all come together to enable people to work effectively in cross-cultural situations (23, 24). In practice, exhibiting cultural competence when communicating with animal guardians accessing services promotes inclusion and collaboration, which leads to higher client satisfaction and improved animal well-being (24). Research on low-income-client-only clinics illustrates that transportation, financial hardship, and care provider-client communication were common barriers, impacting the pet guardian's experience in accessing services (22). Furthermore, research has also demonstrated better service outcomes of using trauma-informed practices (TIP) to serve marginalized populations experiencing various traumas (25, 26). In a service context, a trauma-informed provider realizes the widespread impact of trauma and understands potential ways for healing; recognizes the signs and symptoms of trauma in staff, persons accessing animal services, patients, residents, and others involved in the system; and responds by incorporating knowledge about trauma into policies and practices. This is important because experiencing low-income status is considered a marginality and low-income communities are disproportionally affected by trauma (27).

Kogan et al. (22) argue it is not ethically acceptable to deny families the benefits of a pet due to financial barriers in accessing veterinary health care. Similarly, it has been stated that the lack of access to veterinary care threatens pets and their families (17). Through quantitative survey data from Kogan et al., affordable and accessible veterinary care that results in a positive experience is indicated to improve animal welfare and prevent animals from prolonged distress. Based on this data, they hypothesize that low-income pet guardians are more likely to continue to seek out assistance in the future (22). Previous findings also suggest that a positive experience should involve good communication, be culturally competent, and be relationship-centered with balanced power between the client and veterinarian based on mutuality, negotiation, and joint agreement (23, 28–34).

When discussing veterinary services, it is also essential to consider the stresses on veterinarians. Past studies (35–41) have demonstrated the challenges veterinarians face, including debt, shortage of other veterinarians/large client loads, and emotional challenges due to the impact of working with animals and clients in distress. The COVID-19 pandemic impacted veterinarians' ability to provide services to all clients (13, 14, 18).

Although people's experiences of accessing free or low-cost community veterinary services were measured in the United States (22), there is a scarcity of research that qualitatively describes the experiences of low-income clients accessing private veterinary service with external financial support from animal service agencies. Additionally, studies rarely focus on this issue within the Canadian context. Research dedicated to exploring this context is vital because Canada has a comparatively smaller population and many smaller communities distributed across a wide geographic range, with differing political, health, and social systems. Thus, this study qualitatively examines the COVID-19-driven challenges that low-income pet guardians faced in accessing veterinary care from private veterinary clinics within the Canadian context. We further provide recommendations for improving veterinary and animal services based on the participants' suggestions, informed by their lived experiences and diverse circumstances.

## Materials and Methods

A phenomenological approach was employed to understand low-income pet guardians' experiences accessing veterinary service and their related impacts during the first wave of COVID-19. The details about these experiences were gathered through in-depth, semi-structured telephone interviews. A purposive sampling strategy was utilized to recruit 12 companion animal guardians who lived in Metro Vancouver, British Columbia, Canada and received Companion Animal Veterinary Emergency Funds (CAVEF) provided by VHS. CAVEF receivers were previously screened and identified as low-income according to the Low-Income-Cut-Offs (LICO) chart available from Statistics Canada (42). VHS randomly contacted 29 CAVEF receivers and the first 12 receivers who self-identified their eligibilities were interviewed. Verbal consent was obtained from each participant at the beginning of the scheduled interview. This study was approved by the Social Sciences and Humanities Research Ethics Board at Dalhousie University (certificate number: 2020-5371).

Two of the authors completed the 12 audio-recorded individual telephone interviews over 5 months (from December 2020 to May 2021). The interviews, ranging from half an hour to 1 h, consisted of 14 open-ended questions, which covered topics such as the participants' basic demographic information, their COVID-19-related challenges, and the resources and support they identified and received to address these challenges. The interview protocol (including interview questions) can be accessed from the online data repository of DesignSafe-CI (43). The 12 interviews were transcribed and coded through a content analysis using the qualitative analysis software NVivo 12. The first two authors applied an inductive approach to analyze all the interview transcripts independently. They compared, discussed, and merged their findings into three main subcategories strongly associated with participants' low-income status.

## Results

All participants indicated that their low-income situation was negatively affected by COVID-19 (e.g., a period of limited or no work during the pandemic). This was compounded with other factors that already contributed to their low-income status pre-COVID-19, including having physical or mental health challenges, disabilities, and having existing debt.

### The Barriers to Accessing Veterinary Care Pre- and Peri-COVID-19

The participants identified various barriers. Due to limited appointments, several participants (interviews 1, 4, 5, 6) had to access emergency vet services, which were much more costly than a regular visit. Participants (interviews 1, 6, 8) also shared about the stress of accessing veterinary care. One participant (interview 8) shared, “I have found with COVID [it is] annoying trying to find rides now and I don't like taking my cat in a cab because he's very, very loud.” Typically, they would have found rides with friends, but COVID-19 made that problematic. The limited appointment options were taxing on participants because it was difficult to get an appointment, and with the uncertainty of COVID-19, veterinarians offered restricted hours (interviews 1, 2).

Several participants illustrated a lack of empathy from veterinary workers (interviews 2, 3, 5, 6, 9, 12). Specifically, some participants communicated that despite experiencing low-income, they wished to access services from a veterinarian who could offer affordable quality care (interviews 2, 3, 6, 8). Some felt that veterinarians were overlooking issues with their pets, being short and quick during the visit (interviews 2, 5, 6), and recommending services that the guardian was wary of (interview 12), in one case, leading to the sudden death of a pet (interview 3). Some participants shared experiences that indicated they had to decide out of necessity and affordability, including which clinic they go to (interviews 2, 5). Participants stated that “it seems like they just want the money” (interviews 3, 5), or that there is “inconsistency in pricing and care” (interview 9), that they try “to charge me for things unnecessarily” (interview 12), and described having gone to a vet “where they obviously do not really like animals” (interviews 6, 12). Some participants described needing to see multiple veterinarians to get a second opinion because of this, further exacerbating their state of low-income (interviews 1, 5).

Other barriers mentioned by participants included limited access to financial support when payment was required (interviews 2, 6, 7, 10). One participant stated concerns over the veterinarian keeping an animal in distress due to cost, suggesting: “The veterinary clinic, I think they should be more forgiving on asking for an $800 deposit. Most people especially with COVID don't have that kind of money…[it would be helpful to] work out [a] payment plan or if somebody's helping fund it…that they can wait 'til the next day or a couple days just to be more helpful that way. It's more for the animal, they shouldn't be gatekeeping that care.” (interview 6)

In addition to the cost, the experience of a pet needing emergency care created acute emotional stress for some participants. One participant described the emergency pushing them to their limit: “They had to do a urine test and then a few other things and it ended up being $450 that I just didn't have and we'd already spent so much money on him.” (interview 2) Another participant spoke of themselves and their peers, saying “Everything's fine and all of a sudden bam right? …You just never know. Something goes on with their pets out of the blue and they're not expecting it and everybody's just struggling so hard right now.” (interview 1)

The stress of the appointment was also a challenge. One participant (interview 7) remarked, “I don't have [a] cell phone. So you go … to the vet you drop your pet off and then they call you on your phone while they're doing the exam.” This participant had to find a way to access a phone to communicate with the veterinarian. Another participant (interview 10) shared, “I still have the fear if you can't pay for the bill, they may ask you to surrender the animal and I didn't want to surrender the animal. I can feed her. She's loved. She's not abused.”

### The Emotional Impact of Compounding Barriers Related to Accessing Veterinary Care Peri-COVID-19

Compounding factors created significant stress for low-income pet guardians. These included having essential bonds with their pets that supported their health and the emotional impact of their pet being sick, the emotional and financial stresses of COVID-19, and the impacts of COVID-19 on existing barriers to accessing veterinary care that people experiencing low-income status already face. While the low-income pet guardians interviewed demonstrated resilience by accessing financial and emotional support, they still faced challenging situations.

Participants (interviews 1, 7, 10) noted the emotional impact of the pandemic, primarily in response to the factors that impacted their or their pets' health, such as infection risk in taking transportation. One participant (interview 7) shared, “I was afraid to take a cab because I have three autoimmune diseases.”

Participants indicated the difficulty of choosing between themselves and their animal suffering (interviews 2, 3, 5, 6). A participant (interview 2) stated, “I'd rather go hungry than be able to have my cat die” and “people live under the constant stress because of bills and then having a sick animal…[you] never [want to] be put in a situation that you have to question your animal's health or life over being able to afford a roof over your head.” Similarly, another participant (interview 6) shared their perspective on their own and other low-income pet guardian's experiences: “Nobody should have to choose between paying rent and for veterinary care. I find that a really scary thought.”

The negative mental impact of not participating in veterinary appointments was also tangible for participants (interviews 6, 8). One participant (interview 8) shared: “Not being allowed inside the vet … it's very heartbreaking to not be able to be there with them, [not knowing] what's going on or [being able to] hang out with them because he hates the vet of course.” They felt the phone process created complications in understanding the situation: “I definitely spent a lot more time on the phone going over things with the vets … I feel like it's harder to communicate over the phone.” Another participant (interview 6) also struggled with not being able to comfort their pet, which was difficult for their pet and their mental well-being: “The problem I found was not being able to go in with him 'cause he wasn't used to going to vets, so it was scary for him. …That was a horrible night… honestly, that was really tough.”

### The Human-Animal Bond and Resilience in the Context of the COVID-19 Pandemic

Most participants in this study demonstrated a meaningful human-animal bond, as previous research showed that participants' love for their pets was strong (1). Participants (interviews 3, 5, 6, 7, 8, 10, 11) indicated this as “I love my cats with all my heart and soul” (interview 3), “I've never had a connection to an animal like this” (interview 8), and “She brings us so much joy” (interview 10).

Participants also showed resilience and strength in identifying assistance for their pets (interviews 2, 3, 5, 6, 7, 10, 11). One participant (interview 2) commented about resourcefulness, saying, “I think anybody who's… lived in poverty already knows how … resourceful you have to be.” A participant (interview 3) who collected bottles to help supplement her income to provide food and care for her pets stated, “If it wasn't for me going out collecting those empty bottles I wouldn't have groceries and I wouldn't have gas for my vehicle either.”

Resourcefulness also presented itself as accessing supports from family, friends, and the community (interviews 1, 5, 7, 8, 12). One participant (interview 1) assisted their son with accessing discounted veterinary services and taking his cat to the vet, which was otherwise difficult due to his mental health challenges that COVID-19 exacerbated.

Another participant (interview 7) was able to find support from a friend to overcome the barrier of transportation: “I asked if a good friend of mine would help us, take us to the vet and let me use his cellphone and he let me put coins in the meter, but he wouldn't take any money. [That] was amazing [for] him to do because these vet visits were like 45 min on the phone, right? You can't really go anywhere for coffee or do anything.”

One participant (interview 12) contacted 16 different agencies by doing online searches. “There was quite a few that were independent women that just this is their passion. So they couldn't actually do anything for me other than emotional support, but it was kind of nice for that. And then others, they gave me lists of possible non-profits, that would be able to help and to contact. It was kind of a network that became something that wound up helping me out quite a bit.”

Participants (interviews 2, 3, 7, 10) also demonstrated a willingness to rescue animals in need. Previous research (44) shows the value of rescue for seniors who identify as low-income. One participant (interview 2) stated, “a lot more people who live in poverty or are low-income are more willing to rescue animals, because there's this greater sense of community. You see that a lot too where people who are poor are more likely to be giving to homeless people and give them money. People who are poor will take on animals that have health problems or you know have special needs to help take care of them because of that level of compassion.”

Deeply affected by the pandemic, some participants (interviews 1, 4, 7, 8) began to consider pre-preparedness, especially financial readiness for the next extreme event. One participant (interview 4) shared that they would like to purchase medical insurance for their pets and indicated that their limited income might not support the monthly insurance payment. Another participant (interview 1) proposed that animal clinics could offer some payment flexibility for low-income pet guardians. These factors demonstrate that people experiencing low-income are well-positioned to continue caring for their pets under a service framework that is supportive in addressing social inequities.

## Discussion

Recognizing the challenges low-income guardians and veterinarians faced during the pandemic and the strength of the human-animal bond, this section reviews the participants' recommendations, providing ideas for how veterinary clinics and animal service providers can implement these in their practices.

The most prominent theme mentioned by participants (interviews 2, 3, 5, 6, 9, 12) was more compassion toward low-income pet guardians. Animal and veterinary service providers can work toward providing a trauma-informed model (25) to overcome unconscious bias when providing services to clients that identify themselves as low-income. As specified in the literature, a trauma-informed model is beneficial for the person accessing services and the workers in these circumstances. It leads to better service outcomes by centering a non-judgmental, collaborative, and empathetic approach (26). Resources for trauma-informed care training are in the process of being developed for the animal services sector by the Vancouver Humane Society (25).

One participant (interview 1) suggested accessing more freely available information on assessing their pet's well-being or degree of suffering, sharing that no matter what they ask on the phone, they are instructed to bring their pet in, which can be a significant barrier. Phone conversations or telemedicine to triage an animal, as well as written guidance by email or as a handout as a follow-up to a visit, could provide opportunities to improve access to care and share knowledge in a way that could have a lasting effect and reduce the animal's current and future suffering. Some community-based animal service organizations distribute information (e.g., informative flyers) to pet guardians about animal care; the veterinary sector could expand on this.

Cost, as expected, was a significant barrier to low-income pet guardians accessing services. Participants shared suggestions related to improving access to discounted services. These included reducing limits on charitable veterinary assistance programs, including geographic barriers (interview 2) and the number of animals assisted per person (interview 3), providing assistance with other types of pet services such as pet products (interview 2), providing support for preventative services in addition to emergencies (interview 12), and improved program design as it relates to making programs more accessible (interview 6). Participants also suggested increasing the advertising of programs (interviews 2, 7, 8, 9, 10), suggesting that veterinarians could be aware of veterinary assistance programs and refer clients to them when they share about their state of low-income.

Participants spoke about making payments more feasible, suggesting that veterinarians could offer lower costs (interviews 1, 7) for low-income pet guardians and offer them the opportunity to pay off services over time (interviews 1, 2, 4, 8). A payment plan might not only reduce low-income pet guardian's financial stress but also releases their immediate mental stress, contributing to their overall well-being. Although payment plans and compassionate pricing are not feasible for all private veterinary operations, large-scale veterinary providers that benefit from economies of scale may be able to offer more flexible pricing and payment options. Veterinary clinics can also consider using a spectrum of care treatment or incremental care options to increase access to care for low-income animal guardians (45). For more recommendations related to cost, Mattson compiled suggestions for veterinarians to better provide access-to-care options during the COVID-19 pandemic (46).

Participants (interviews 4, 9, 12) also suggested that prices could be regulated between veterinarians and the government authorities (interview 2) in providing support that recognizes the mental health benefits of the human-animal bond for low-income individuals. This points toward the role that regulatory bodies and government can have in supporting low-income pet guardians.

Collaboration, collective decision-making, and compassionate care go a long way in establishing trust, so does having cultural competence (24). Trust leads to better understanding and compliance, resulting in a better animal welfare outcome. As demonstrated through the findings, [Kogan et al. (22), p5] expertly outline, “pet owners who feel respected and heard are more likely to seek out care and follow medical recommendations.”

Veterinary clinics and animal service agencies can also benefit from this positive experience. They may feel more understanding and receive more kindness from clients. Low-income pet guardians may have limited prior veterinary medicine experience. For example, Wiltzius et al. (17) found that nearly 1 out of 4 respondents in their study, who were disproportionately low-income, shared that they were unable to access preventative veterinary care for at least one of their pets in the recent past, and faced this barrier at an average frequency of 2.4 times in the past year (17). This emphasizes the importance of each visit being a positive experience such that veterinary care is valued and prioritized in the future.

Another benefit that veterinary and other animal service providers may experience is that animals are likely to come in sooner when there are subtle signs of being in need rather than later when the situation may be at a crisis point. Seeing the animals when an issue first occurs may decrease the likelihood of euthanizing animals for reasons related to their owner's financial status, which can take an emotional toll on veterinary workers.

## Conclusion

This study explored the experiences of low-income pet guardians regarding accessing veterinary care during the COVID-19 pandemic. The study found that participants who experienced pandemic-related barriers that were related to and compounded by their low-income status can be categorized in three aspects: the barriers to accessing veterinary care before and during COVID-19, the emotional impact of compounding barriers related to accessing veterinary during COVID-19, and the human-animal bond and resilience in the context of the COVID-19 pandemic.

The global COVID-19 pandemic has created an opportunity to evaluate existing support services, especially those programs that were developed for low-income pet guardians. To prepare for future disaster situations, this study suggests that animal services and veterinary clinics could increase their financial capacity to support people needing assistance, undergo training to learn how to better work with people experiencing financial and emotional stress, and gather more information and resources that can be easily shared to better distribute knowledge about animal needs and available financial assistance programming. From a One Health and One Welfare perspective, these recommendations could positively impact pet guardians, their pets, and the service providers.

## Data Availability Statement

The datasets presented in this article are not readily available because of privacy and ethical concerns. The interview data will be shared at reasonable request to the corresponding author. Requests to access the datasets should be directed to Haorui Wu, Haorui.wu@dal.ca.

## Ethics Statement

The studies involving human participants were reviewed and approved by Social Sciences and Humanities Research Ethics Board at Dalhousie University (Certificate No: 2020-5371). The participants provided their verbal informed consent to participate in this study.

## Author Contributions

AM and HW conceived the study design, analyzed the data, and drafted the manuscript. CM conducted interviews and edited the manuscript. All authors contributed to the article and approved the submitted version.

## Funding

This research was supported by the Social Sciences and Humanities Research Council (SSHRC) Canada, Partnership Engage Grants (Award # 1008-2020-0246). This research was also undertaken, in part, thanks to funding from the Canada Research Chairs (CRC) Program (Award # CRC-2020-00128).

## Conflict of Interest

AM and CM are the employees of Vancouver Humane Society. Neither of them received financial support to conduct this research. The remaining author declares that the research was conducted in the absence of any commercial or financial relationships that could be construed as a potential conflict of interest.

## Publisher's Note

All claims expressed in this article are solely those of the authors and do not necessarily represent those of their affiliated organizations, or those of the publisher, the editors and the reviewers. Any product that may be evaluated in this article, or claim that may be made by its manufacturer, is not guaranteed or endorsed by the publisher.
